# Local dynamic mechanical analysis for heterogeneous soft matter using ferrule-top indentation†
†Electronic supplementary information (ESI) available. See DOI: 10.1039/c6sm00300a



**DOI:** 10.1039/c6sm00300a

**Published:** 2016-02-24

**Authors:** Hedde van Hoorn, Nicholas A. Kurniawan, Gijsje H. Koenderink, Davide Iannuzzi

**Affiliations:** a Department of Physics and Astronomy , VU University , De Boelelaan 1081 , Amsterdam , The Netherlands . Email: h.van.hoorn@vu.nl ; Email: d.iannuzzi@vu.nl; b Laserlab Amsterdam , VU University , De Boelelaan 1081 , Amsterdam , The Netherlands; c FOM institute AMOLF , Science Park 104 , Amsterdam , The Netherlands

## Abstract

There is a strong demand for nanoindentation methods to probe the heterogeneous viscoelastic properties of soft tissues. Important applications include diagnosis of early onset diseases such as arthritis and investigations into cellular mechanoresponse in tissue. Quantification of tissue mechanics at length and time scales relevant to biological processes, however, remains a technical challenge. Here, we present a new nanoindentation approach that is ideally suited to probe the viscoelastic properties of soft, hydrated tissues. We built a ferrule-top probe that uses wavelength modulation in a Fabry–Pérot cavity configuration to detect cantilever deflection and to drive a feedback-controlled piezoelectric actuator. This technique allows us to control the static load applied onto the sample using an all-optical mm-sized probe. We extract the local elastic and viscous moduli of the samples by superposing a small oscillatory load and recording the indentation depth at the frequency of oscillation. By using a set of silicone elastomers with a range of stiffnesses representative of biological tissues, we demonstrate that the technique can accurately determine moduli over a wide range (0.1–100 kPa) and over a frequency range of 0.01–10 Hz. Direct comparison with macroscopic rheology measurements yields excellent quantitative agreement, without any fitting parameters. Finally, we show how this method can provide a spatially-resolved map of large variations in mechanical properties (orders of magnitude) across the surface of soft samples thanks to high sensitivity over large (>μm) cantilever deflections. This approach paves the way to investigations into the local dynamic mechanical properties of biological soft matter.

## Abstract 2

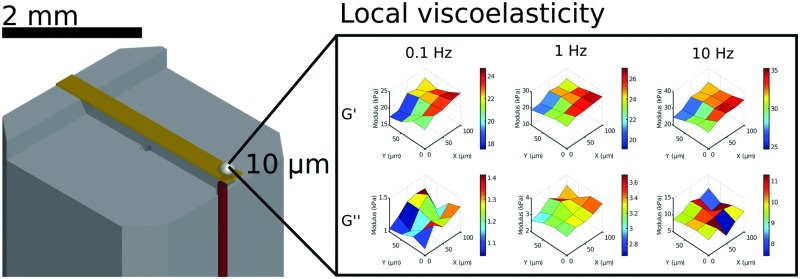
We present a mm-sized, all-optical probe that can accurately quantify and map the viscoelastic properties of highly heterogeneous soft matter.

## 1 Introduction

The mechanical properties within biological tissues are highly diverse and impact a wide range of physiological processes.^1–3^ Tumor growth and the related process of angiogenesis have for instance been shown to be influenced by the extracellular niche in which tumor cells reside.^4,5^ The differentiation of stem cells is also sensitive to the stiffness of their extracellular environment.^6,7^ In general, cells function in a protein-based network known as the extracellular matrix, whose main constituent is collagen. This network in itself already has a complex hierarchical molecular composition across nano- to micrometric length scales.^8^ In tissues, embedded cells (that actively exert forces onto the matrix^9^) further complicate the overall mechanical response.^10,11^ Therefore, the mechanical properties of tissues are very difficult to characterize experimentally, especially at micron length scales where both network complexity and cellular mechanics play a role.

Tissue mechanics has traditionally been probed by macroscopic mechanical tests such as stretching and shearing.^12^ These tests have revealed interesting mechanical features such as strain-stiffening and active stiffness control by cells. However, since they average over large length scales, these methods cannot provide insight into the properties of the local niche surrounding the cells. Cells are sensitive to mechanical properties of tissues at scales comparable to their size, which is typically 10–100 μm.^13^ Cells physically connect to the extracellular matrix *via* adhesion plaques that have a size between 0.1 and 5 μm.^14,15^ Relevant heterogeneity of the local environment influencing cellular processes thus occurs at micrometer-length scales.

In recent years several approaches have been developed to quantify local mechanical properties in tissues. It is important to note that tissues have significant time-dependent mechanical behavior, which necessitates a rheological approach to quantify mechanics. A class of rheology that measures local (micron-scale) properties is termed microrheology, which can be performed by either following spontaneous, thermally driven fluctuations of micrometer-sized beads, or by actively driving them with optical or magnetic tweezers.^16,17^ Microrheology has the advantage of being sensitive and suitable for soft samples.^18^ However, tissues are generally too stiff for this approach, since the force range available with thermal fluctuations or even optical and magnetic tweezers is too low to yield measurable deformations.^19^

Atomic force microscopy (AFM), on the other hand, can reveal interesting dynamic mechanical properties of biological matter,^20–22^ but only a limited range of stiffnesses can be probed with a single cantilever. The origin of this limitation lies in the limited cantilever deflection that can be measured using AFM. Cantilever deflection in AFM is typically quantified using optical beam reflection using a split or quadrant photodiode. One can also employ single-wavelength interferometry and obtain similar sensitivity,^23^ using the linear regime around quadrature (see Experimental). However, to map surfaces that are highly heterogeneous in stiffness the limited detectable cantilever deflection range poses an upper and lower limit on the detectable stiffness for a certain indentation depth (which is also limited by the linear deformation regime, see Discussion, ESI†). Thus, it remains a technical challenge to measure micrometer deformations with large cantilever deflections at high sensitivity to quantify large heterogeneities in stiffness across soft tissues.

Tissues and cells indeed have varying elastic moduli in the order of 0.1–100 kPa.^2,24^ Over this stiffness range, a cantilever will deflect over three orders of magnitude for a given indentation depth. At a stiff location the deflection will be larger than in a soft location. However, in practice a high sensitivity is also required to quantify the low stiffnesses typical for a biological sample. One thus needs high sensitivity over a micrometer-range of cantilever deflection to quantify tissue heterogeneity.

Moreover, previous studies mostly assume quasi-static elasticity in tissues and cells.^1,2,24^ This crucial assumption implies that the measured elastic moduli do not depend on deformation rate. However, this is often not the case for biological materials. Brain tissue, for instance, shows a change in both viscous and elastic moduli depending on the rate of deformation.^25^ Even the purified constituents of both extracellular matrix^26,27^ and intracellular cytoskeleton^28,29^ already show strain rate-dependent stiffening. Furthermore, forces exerted by individual cells are on the order of 1–10 nN with deformation of the matrix on a timescale of seconds,^15,30^ again influencing dynamic mechanical properties in tissues. All these effects occur in a frequency range of 0.01–10 Hz and also need to be taken into account to quantify tissue viscoelasticity.

To address the challenge of measuring the heterogeneous viscoelastic properties of tissues, we demonstrate a new approach. We recently showed that ferrule-top probes can be used as a miniaturized atomic force microscope with robust calibration of bending stiffness.^31–33^ Here, we demonstrate a new approach to dynamic measurements capable of quantifying a wide range of stiffnesses, in a miniaturized and versatile all-optical probe. We apply wavelength-modulated Fabry–Pérot interferometry with a lock-in amplifier, live demodulation and a feedback-loop on probe movement. This enables us to apply a controlled load or indentation depth onto a sample and then probe the dynamic mechanical response at frequencies of 0.01–10 Hz. To benchmark our technique, we quantify the dynamic response at the micrometer-scale of silicone polymers that are often used as substrates for cell studies^34–38^ with a measured precision of 5–15%. Moreover, without any fitting parameters, our independent characterization agrees very well with macro-rheology measurements over a broad range of stiffnesses. Our local quantifications accurately follow the macro-rheology frequency-stiffening trend for shear moduli ranging over 0.1–100 kPa. Finally, we demonstrate that it is possible to map local stiffness variations over more than one order of magnitude. Our mm-sized all-optical probe can thus measure cantilever deflection over a large range to map highly heterogeneous local mechanical properties.

## 2 Experimental

**Fig. 1:**
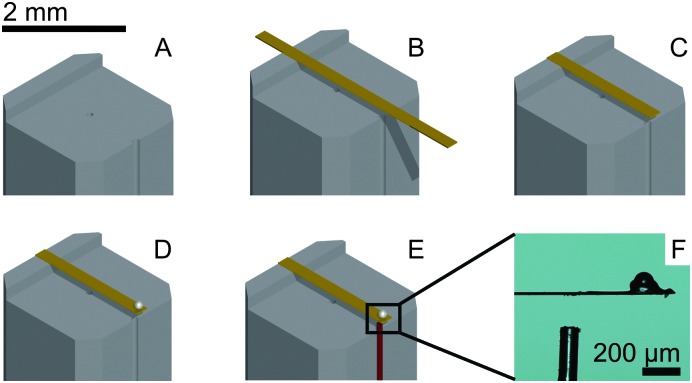
Ferrule-top probe fabrication. A ridge is cut and an Au-coated cantilever is glued (A and B) on a borosilicate ferrule. The cantilever is cut to size (C) and a glass bead is glued to the tip (D). A single-mode fiber (in red) is positioned (E) to form an interferometric cavity of approximately 200 μm (F) over which cantilever deflection is measured.

The process of probe fabrication is schematically depicted in Fig. 1. Ferrule-top probes were fabricated from 3 × 3 × 7 mm glass ferrules (CM Scientific) micro-machined on a wire-cutter (Well Diamantdrahtsagen GmbH) to make a ridge and a groove to position a single-mode optical fiber (Corning SMF-28, step A). Glass ribbons (Vitrocom), sputter-coated with a 100 nm Au layer, with cross-section 20 × 200 μm or 30 × 300 μm, were then placed on the ridge (step B). The ribbons were then cut to the desired length (to control the bending stiffness), and a bead was fixed to the cantilever tip (steps C–D). Finally, the probe was mounted on a holder for the piezoelectric actuator and a single-mode fiber was mounted in the groove (step E). The interferometric cavity between the fiber-air and air-cantilever interfaces was set at approximately 200 μm (microscopy image in Fig. 1F). Double reflections in the interferometric cavity were avoided by exposing the optical fiber tip to a brief plasma arc before mounting it under a ∼3° angle relative to the cantilever.

**Fig. 2:**
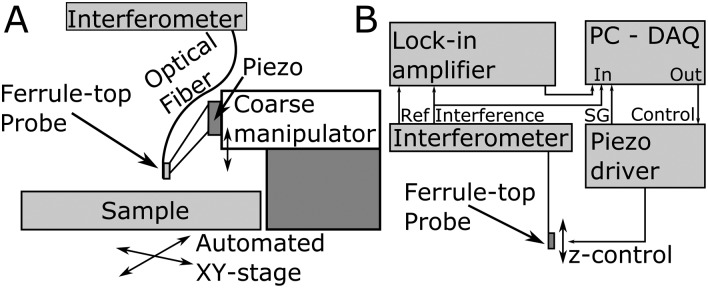
Schematic of the (A) indentation setup and (B) readout and feedback control. (A) The ferrule-top probe is mounted on a long-range piezoelectric actuator and a coarse manipulator. The optical fiber is attached to an interferometer and the sample can be scanned in plane (*XY*) between indentations. (B) High-frequency wavelength modulation is applied onto the infrared light source and the interference readout and the lock-in signal of the corresponding amplitude enable a large-range readout of cantilever deflection. Feedback control onto the piezoelectric driver relative to the required deflections enables load-controlled indentations using the ferrule-top probe.

The probe was mounted on a long-range piezoelectric manipulator (P-602.5SL, 500 μm range, Physike Instrumente GmbH), which in turn was attached to a manual *z*-manipulator (see Fig. 2A). The piezoelectric actuator was feedback-controlled by means of a direct readout of the actual extension through a strain gauge. We placed the sample on a motorized *XY*-stage (AG-LS25, Newport) with a USB-controlled driver for automated surface scanning. The single-mode optical fiber from the ferrule-top probe was then connected to an interferometer with a tunable laser source around *λ* = 1550 nm (OP1550, Optics11^39^).

The interferometric readout measure of cantilever deflection *d* typically relies on the photodiode voltage scaling linearly for small deviations around quadrature 
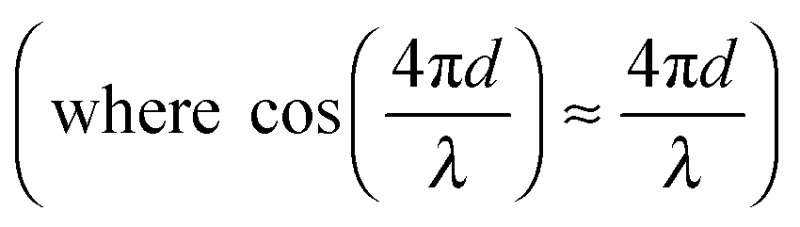
.^23,31^ However, around quadrature the range of cantilever deflection readout is then limited to ≈*λ*/8. The full interferometric readout (over multiple periods of constructive and destructive interference) is given by:1
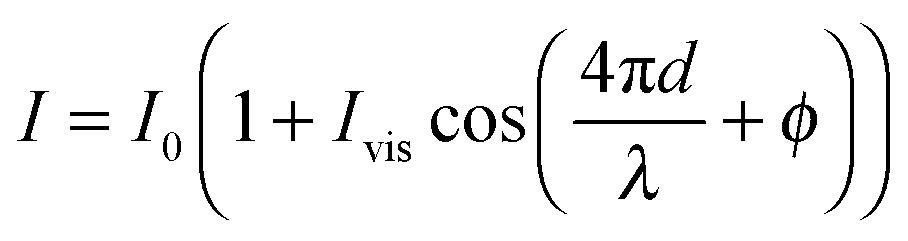
where the response of the direct interferometric readout *I* is given as a function of wavelength *λ*, cavity distance *d*, visibility *I*_vis_ (=(*I*_max_ – *I*_min_)/(*I*_max_ + *I*_min_)) and offset *I*_0_ (=(*I*_max_ + *I*_min_)/2). However, the linear range of voltage *versus* cantilever deflection is fundamentally limited by the wavelength (with *λ* = 1550 nm the linear range is approximately 200 nm). In our setup, we were able to enlarge the range of cantilever deflection readout by using wavelength modulation and a lock-in amplifier (see Fig. 2B). The source wavelength was sinusoidally modulated at 10 kHz with an amplitude of ∼100 pm. The reference frequency (10 kHz step function) and the interferometric signal were then sent to a lock-in amplifier (SR830, Stanford Research Systems). The amplified amplitude of the high frequency signal was used as analog input on a Data AcQuisition (DAQ) card (PCIe-6361, National Instruments) next to the direct interferometric readout and the strain gauge readout. The displacement of the piezoelectric translator was driven through an analog output from the same DAQ card. Data readout, processing and instrument control were performed in custom-written Labview (National Instruments) software.

The effect of wavelength modulation on the interferometric intensity signal (with *φ* = 0) is given by:2
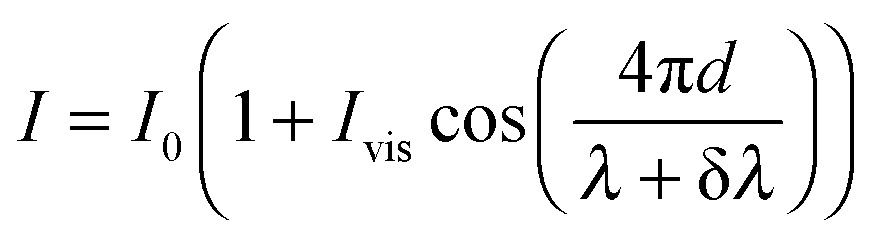



**Fig. 3:**
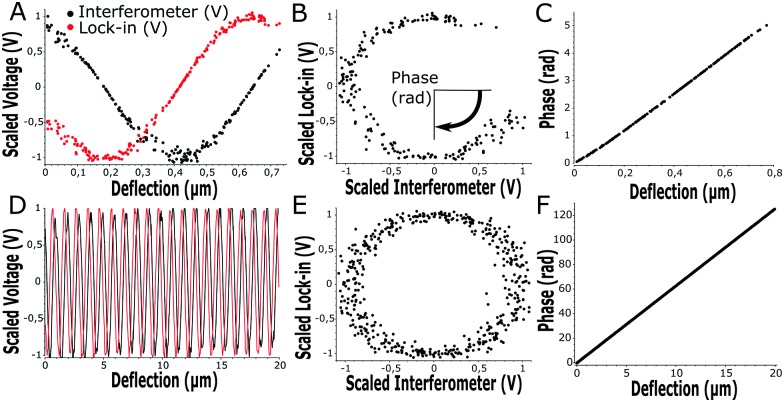
Wavelength modulation for high sensitivity with small (A–C) and large (D–F) cantilever deflection. (A and D) Interferometer- (black) and wavelength-modulation amplitude from the lock-in amplifier (red) show constant visibility over large cantilever deflections with a π/2 phase-shift. (B and E) Scaled interferometer and lock-in signals give a direct relation to cantilever deflection as the angle through the circle given by interferometer and lock-in signal (phase in rad). The unwrapped angle shows a linear relation to the deflection with high sensitivity over small (C) and large (F) deflections.

With a sinusoidal modulation of δ*λ* = Δ*λ* sin(*ω*_mod_ *t*) the interferometric response can be approximated through a Bessel- or a Taylor expansion (both yield the same first-order approximation) around Δ*λ* = 0 by:3
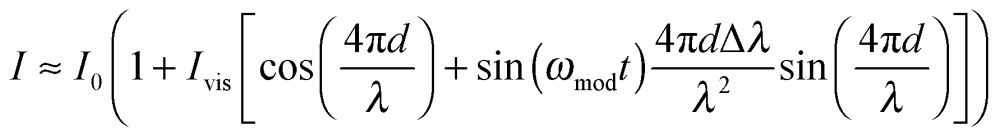
where this approximation shows (1) a low-frequency component similar to the normal interferometric response that scales with 
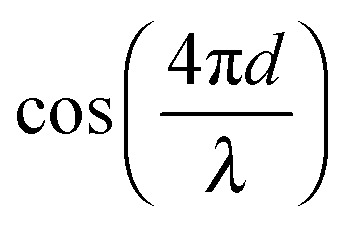
 and (2) a high-frequency component with amplitude scaling with 
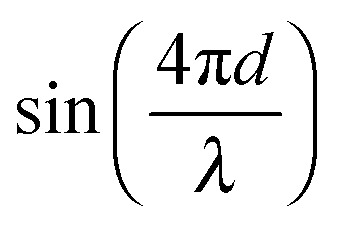
. The low-frequency response was measured as the direct interferometric readout, while the high-frequency amplitude at the modulation frequency was given by the lock-in amplitude. Over a full period of 
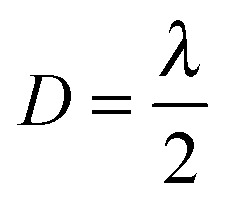
, we obtained a unique solution for the cantilever deflection *d* as a function of interferometric voltage and lock-in voltage. Since the cantilever did not deflect more than 
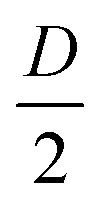
 within our sampling time, we could apply phase unwrapping (*i.e.* keep track of the number of periods). Finally, we obtained the cantilever deflection in principle indefinitely, but in practice as long as the visibility of the scaled interferometric and lock-in signal remained constant over deflection (see Fig. 3).

Indentations were always performed in the linear viscoelastic regime. A more extensive theoretical consideration is presented in the ESI.† Briefly, we first performed a regular quasi-static stiffness measure to check if the desired load and indentation depth were attainable. We then defined an oscillatory load-sweep over time to be performed on top of a static load, all within the apparent linear elastic limit. When mapping highly heterogeneous samples it is crucial to control either load (*i.e.* cantilever deflection) or indentation depth, to ensure consistent stress or strain, respectively. If only the probe movement were controlled, the indentation depth would be much lower on a stiff- compared to a soft location. As a consequence, the assumptions that we are in the linear viscoelastic regime or that indentation depth is much smaller than bead radius (*h* ≪ *R*) may no longer be valid. To provide load- or indentation-depth control, we performed feedback using an error signal. The error signal from the load sweep and the continuously measured load *e*(*t*) = *P*_sweep_ – *P*_measured_ was then input into a feedback loop with action onto the extension of the piezoelectric actuator. The indentation depth also depends on the cantilever deflection and follows from *h*(*t*) = *d*_piezo_ – *d*_cantilever_. With a predefined indentation sweep the error signal *e*(*t*) = *h*_sweep_ – *h*_measured_ similarly produced a dynamic indentation sweep.

Using the load *P* and indentation depth *h* over time we measured the dynamic mechanical moduli at distinct frequencies. We employed an analytic solution obtained in previous research on oscillatory nanoindentation to obtain the storage- and loss moduli.^40^ There was no indication of plastic deformation in our samples and the load-indentation curve showed a Hertzian trend (Fig. S3, ESI†). Therefore, we employed a theoretical description for smooth sphere-surface contact.^41^ The theoretical description uses Sneddon's relationship which translates stress and strain to load and indentation depth.^42,43^ The beads were spherical and the surfaces were flat with respect to the micrometer-scale indentations. Surface roughness on the scale of indentation was insignificant relative to the deformations, while the point-of-contact with the surface in individual indentations was determined by using a threshold for cantilever deflection. For an oscillatory load and indentation, the equations for shear storage- and loss modulus *G*′ and *G*′′ with a spherical indenter are:4
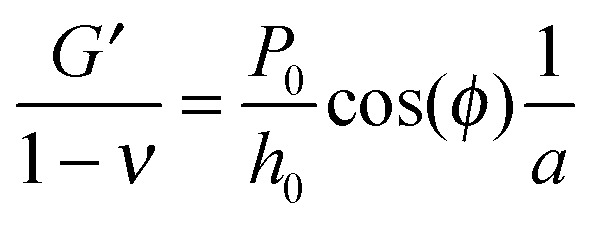

5
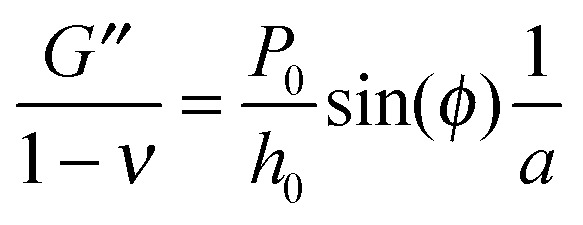
where *P*_0_ is the amplitude of the oscillatory load, *h*_0_ is the amplitude of indentation depth, *ν* is Poisson's ratio of compressibility, *φ* is the phase-shift between load and indentation and *a* is the contact radius. In the absence of adhesion we estimated *a* (as confirmed through finite element modeling^36^) as 
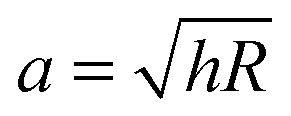
, with the assumptions that *h* = 2*h*_contact_ and *h*_contact_ ≪ *R*. During indentation the sample is both sheared and extended/compressed. Therefore, the quantitative comparison with the shear modulus depends on the compressibility. In a biological sample, one could still measure an effective storage- and loss modulus, but not a shear or Young's modulus directly without knowledge of *ν*.

To test our approach over a wide range of stiffnesses, we prepared two types of silicone elastomer: Sylgard 184 and Sylgard 527 (both from Dow Corning). Sylgard 184 Poly(DiMethyl)Siloxane (PDMS) is often used as a substrate for cells and its stiffness can be varied by changing the crosslinker:prepolymer weight ratio.^34,37^ We used Sylgard 184 samples with 1 : 20 and 1 : 50 ratio, as well as Sylgard 527 at 1 : 1 ratio. Sylgard 527 is much softer than Sylgard 184 and has recently been shown to also function as a cell substrate with tunable stiffness.^38^ The Sylgard 527 sample will be referred to as “Low Stiffness” (LS), the Sylgard 184 1 : 50 as “Medium Stiffness” (MS) and the Sylgard 184 1 : 20 as “High Stiffness” (HS). These silicone polymers are incompressible, *i.e.* their Poisson ratio *ν* = 0.5.^44,45^ All samples were prepared by weighing the compounds in the desired ratios, rigorous manual mixing, degassing in a desiccator and curing for 16 hours at 65 °C. Subsequently, the samples were allowed to cool to room temperature before the experiment (indentation or macro-rheology) was started. To exemplify the surface mapping functionality of our approach, samples with a gradient from 1 : 20 PDMS to 1 : 50 PDMS and from 1 : 20 PDMS to Sylgard 527 were prepared. A barrier for the liquid (uncured but mixed and degassed) PDMS was first added to separate the two components and removed just before the sample was placed in the oven. As the PDMS then cured, the two samples mixed and created a gradient of stiffness where the barrier had been placed.

The MS and LS samples turned out to be very adhesive to our glass sphere probe. We therefore passivated the samples used for indentation by incubating for 30 minutes at room temperature with 5 wt% Bovine Serum Albumin (BSA) in Phosphate Buffered Saline (PBS, both from Sigma Aldrich). All indentation experiments were performed with both the sample and the probe fully submerged in PBS. This demonstrates the applicability of our approach to biological materials, cells, and tissues, in their native, hydrated state.

To provide a quantitative comparison to our dynamic indentation measurements, we used a commercial rheometer (MCR-501, Anton-Paar) to directly measure the macroscopic shear modulus of our samples. We used a cone-plate geometry (with 20 mm cone diameter and 1° or 2° angle) and an oscillatory frequency sweep over 0.01–10 Hz to obtain the macroscopic *G*′ and *G*′′. In the rheology measurements, we applied a uniform shear strain to the sample (unlike in the indentation experiment). We also performed strain-sweeps to confirm that the material was indeed linearly viscoelastic (see Fig. S1, ESI†), as required by the theoretical assumptions. In all reported frequency sweeps, we tested the dynamic response (both in local indentation- and in global rheometer measurements) within the linear viscoelastic limit.

## 3 Results and discussion

At the start of each experiment, the probe was calibrated on a glass surface to obtain the periodicity of the interferometric readout relative to cantilever deflection. For a good probe, the scaled lock-in *versus* interferometer readout showed a signal moving through a circle, where the angle relative to the center scales linearly with probe displacement according to theory (eqn (3) and Fig. 3). If the cavity was exactly behind the point of contact, the calibrated cantilever displacement per angle in rad was equal to 
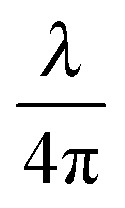
, according to theory (eqn (1)). However, the measured cantilever displacement was often not exactly behind the point-of-contact (as can be seen in Fig. 1F). Calibration on a glass surface included this geometric effect and provided a check for the linearity between unwrapped angle and cantilever displacement (as depicted in Fig. 3C and F). These results immediately demonstrate the strength of this approach: we can quantify cantilever deflection over 20 μm, a 100-fold increase relative to the linear approximation around quadrature.

Instead of defining the movement of piezoelectric extension, we exerted a defined load or indentation depth on the sample using custom-designed Labview software driving the piezoelectric actuator based on the live readout of load or indentation depth. This feedback allowed us to isolate either creep or stress relaxation when the load or indentation depth, respectively, were controlled. On samples with heterogeneous stiffness, at a given indentation depth, we were now able to measure the load-response over a large range thanks to the increased cantilever deflection readout (up to 20 μm, Fig. 3F). With a defined load or indentation depth, we could then accurately quantify both the viscous and elastic response of the sample. To probe viscoelasticity at distinct deformation rates, we defined a sweep of load or indentation-depth over time. The sweep was activated once the indenter made contact with the sample (set by a threshold value dependent on cantilever noise at 4*σ*, see Fig. S2, ESI†).

**Fig. 4:**
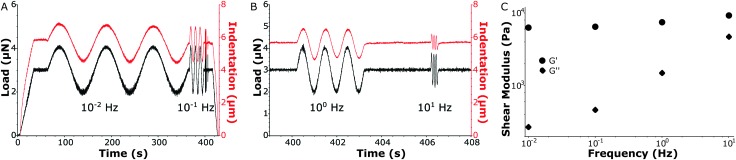
Local measurement of shear storage and loss modulus of a MS sample. (A, fast oscillations of A magnified in B) Oscillatory load (black) with a constant hold load of 3 μN and an oscillation amplitude of *P*_0_ = 1 μN over a frequency sweep at 10^–2^, 10^–1^, 10^0^ and 10^1^ Hz. The corresponding indentation depth (red) measures, at these frequencies, the resulting oscillatory indentation amplitude *h*_0_ and phase shift *φ* between oscillatory load and indentation. The absolute indentation depth determines the contact radius *a*. (C) *G*′ and *G*′′ describe the elastic and viscous responses, respectively, at distinct oscillatory frequencies.

In Fig. 4A and B a load-sweep (black line) with indentation response (red line) is depicted for a MS sample. By continuously adjusting the piezoelectric extension, the predefined load sweep was followed well over time. Typically, the load increased linearly up to a constant value (3 μN in the figure) which was then held to let the sample creep up to an approximately constant indentation depth. Next, load oscillations (in this example at *P*_0_ = 1 μN) were performed at different frequencies (in this example at 0.01 Hz, 0.1 Hz, 1 Hz and 10 Hz, see Fig. S4 (ESI†) for individual frequency fitting). The indentation depth response then characterized the viscoelastic material properties *via* the oscillatory indentation depth *h*_0_, the phase shift between load and indentation *φ* and the contact radius *a*. To quantify *a*, we needed to accurately determine the absolute value of the indentation depth. We therefore fitted the load-indentation curve of approach with the Hertz equation (see Fig. S3, ESI†). Finally, all the fitted parameters quantify *G*′ and *G*′′ as given by eqn (4) and (5), respectively (see Fig. 4C). In subsequent experiments we expanded the frequency-sweep to typically include 20 frequencies over the full range of 0.01–10 Hz on a logarithmic scale.

Using a macroscopic shear rheometer, we performed rheology experiments on identically fabricated silicone polymer samples to validate the results from our dynamic indentation approach. We performed tests with increasing strain to ensure that both the shear rheology and the oscillatory indentations were performed within the linear viscoelastic regime. Macroscopic rheology showed that the linear elastic regime typically extended up to a strain amplitude of about 20% (see Fig. S1, ESI†). A Hertzian fit to the indentation curves indicated the indentations were also within the linear regime (see Fig. S3, ESI†). To demonstrate the large range of stiffnesses that can be probed with our technique, we tested different silicone polymers that span the physiologically relevant range for tissue stiffness (0.1–100 kPa). We compared the macroscopic shear moduli as measured with the rheometer to microscopic moduli as quantified with dynamic indentations.

**Fig. 5:**
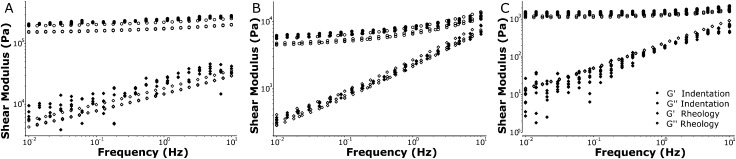
Quantitative comparison of the viscoelastic behavior of silicone polymers. Our dynamic indentation approach (closed symbols) gives quantitatively similar results to macrorheology (open symbols) for both storage modulus *G*′ (circles) and loss modulus *G*′′ (diamonds), over a range of moduli (0.1–100 kPa) for (A) low-, (B) medium- and (C) high stiffness silicone polymer samples. The frequency-dependent behaviour of *G*′ and *G*′′ over a range of 0.01–10 Hz is well captured in local indentations as compared to macrorheology.

Fig. 5 shows the dynamic response of silicone samples with stiffnesses over the frequency range of 0.01–10 Hz. Note that the rheology and indentation approach are fully independent measurements and that no fitting or parametrization was performed to compare the results from both techniques. The HS samples showed predominantly elastic behavior (*G*′ ≫ *G*′′) and a weak frequency dependence of the loss modulus. Our MS samples showed more significant frequency-dependent stiffening (increase in *G*′) and in particular more fluid-like behavior (increase in *G*′′), consistent with our indentation experiments. The LS samples showed little stiffening, and again a large increase in the loss modulus with increasing frequency. Overall the frequency dependence observed using our indentation experiments was accurately recovered in the macroscale rheometry and the absolute values for both storage- and loss moduli corresponded very well. For all samples tested we were able to quantify the local stiffness to a standard deviation of 5–15% relative to the absolute values for *G*′ and *G*′′ in repeated measurements on the same location (see Fig. S5, ESI†). Taken together, these results show that we can accurately measure frequency-dependent properties using local indentations over 3 orders of magnitude in stiffness ranging from 0.1–100 kPa.

**Fig. 6:**
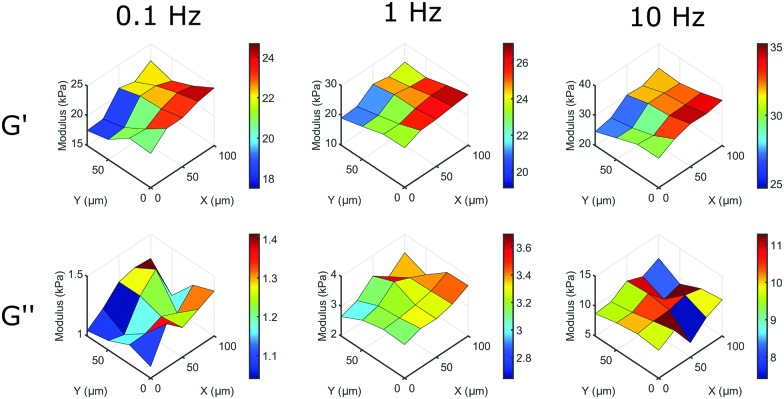
Mapping local mechanical properties on the surface of a HS to MS sample. By scanning the surface of a sample with a premade stiffness gradient we can observe significant variations in local viscoelasticity. On this surface, *G*′ increases significantly in the +*x* and –*y* direction on a length scale of 10 μm at different frequencies, while still showing the overall increase in *G*′ with increasing frequency. *G*′′ shows the overall increase in modulus with frequency, but shows less surface variations (for details on measurement precision, see Fig. S5, ESI†).

The main advantage of our indentation approach over macroscopic methods is the ability to measure local viscoelastic properties. To demonstrate this, we mapped the stiffness on the surface of a silicone sample with a gradient from a HS to a MS sample. On a length-scale of 10 μm we could observe variations in local viscoelasticity. The resulting maps of *G*′ and *G*′′ are depicted in Fig. 6 at 3 different frequencies. The maps of *G*′ (top row) show an increase in elastic response in both +*x* and –*y* direction (left to right in the image) and a higher modulus with increasing frequency, similar to what we observed in homogeneous samples. The viscosity as given by *G*′′ (bottom row) shows less spatial variation, close to our measurement precision (see Fig. S5, ESI†). The absolute values of *G*′′ on the entire measured surface still increase with increasing frequency, as we observed in homogeneous samples. Using our dynamic quantification we can thus investigate soft matter rheology on a micrometer-scale.

**Fig. 7:**
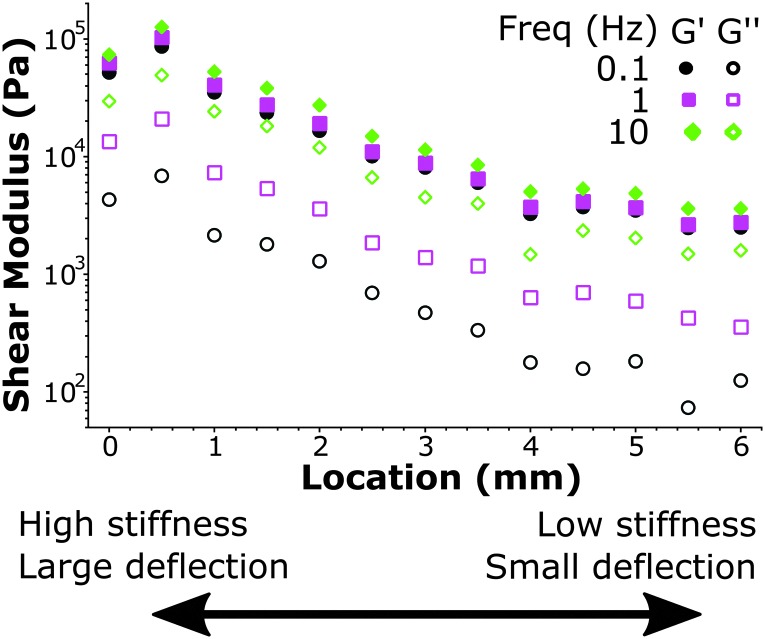
Mapping viscoelastic properties over a large stiffness range on a HS–LS gradient sample. At a high stiffness location (left) the large cantilever deflection (∼20 μm) can be measured while at a low stiffness location (right) the small cantilever deflection (∼200 nm) can also still accurately quantify stiffness. At a controlled indentation depth the material is deformed to the same degree on all locations, while the viscoelasticity is quantified over more than one order of magnitude.

We further demonstrate the ability to map viscoelasticity over a large range of stiffness in Fig. 7. The storage and loss modulus at three frequencies are depicted *versus* location across the boundary of a stiffness-gradient sample from HS to LS. With the large cantilever deflection readout, we were able to measure the viscoelastic properties over more than one order of magnitude in both storage and loss moduli. Since the load scales linearly with bulk modulus, we can measure stiffness variations using this method over 2 orders of magnitude, since we can reliably measure low stiffness locations with cantilever deflections of ∼200 nm and with the same probe measure large phase-unwrapped deflections up to ∼20 μm (as shown in Fig. 3). These results thus demonstrate quantitative viscoelastic mapping of soft matter with stiffness heterogeneity over more than one order of magnitude.

## 4 Conclusions

Here, we present a new approach that opens up possibilities in the investigation of soft and biological matter at length scales of cells and tissues. We measured storage and loss moduli of silicone polymers over a range of 0.1–100 kPa with a frequency range of 0.01–10 Hz. We reproducibly quantified the dynamic mechanical properties that scaled with frequency similarly to the macroscopic shear moduli with a precision of 5–15% relative to the absolute values of the moduli. Using spatial mapping we could further distinguish significant inhomogeneities in stiffness on a micrometer length-scale and quantify large dynamic stiffness variations over more than one order of magnitude. We have thus developed a technique that can probe dynamic mechanical properties at a cellular length scale and at stiffness values relevant to soft biological matter.

It will be of particular interest to use this approach to gain insights into the local mechanical properties of a biological material in relation to its global mechanical response. An even more exciting application of this technique is for measurements of soft tissue samples where the presence of certain cell types and extracellular matrix in varying composition can cause large spatial variations in the local mechanical response. In many of these applications a combined effort with microscopy techniques can simultaneously track network deformation and/or specific cellular/network compositions. Ultimately, we aim to utilize our small all-optical probe in *in vivo* applications.
