# Supplementary material for: Local dynamic mechanical analysis for heterogeneous soft matter using ferrule-top indentation
Source: Soft Matter. 2016 Feb 24;12(12):3066–73. doi: 10.1039/c6sm00300a (PMC4819682; doi:10.1039/c6sm00300a)
Supplement: Supplementary file 1 [file SM-012-C6SM00300A-s001.pdf]

Electronic Supplementary Material (ESI) for Soft Matter.
This journal is © The Royal Society of Chemistry 2016

# Supplemental Information
# Local dynamic mechanical analysis for soft matter using ferrule-top indentation

Hedde van Hoorn[1,2], Nicholas A. Kurniawan[3,4], Gijsje H. Koenderink[1,3], and Davide Iannuzzi[1,2]

[1]Department of Physics and Astronomy, VU University, Amsterdam, the Netherlands
[2]Laserlab Amsterdam, VU University, Amsterdam, the Netherlands
[3]FOM institute AMOLF, Amsterdam, the Netherlands
[4]Department of Biomedical Engineering, Eindhoven University of Technology, Eindhoven, the Netherlands

February 18, 2016

# 1 Linear viscoelasticity

Using a macroscopic shear rheometer, we checked whether all samples behaved as linearly viscoelastic materials in the strain range we probed. As we gradually increased shear strain, both $G'$ and $G''$ remained constant up to $\sim 20\%$ strain for all frequencies probed (see the results for $f = 1$Hz in figure S1).

The cantilever on our probe behaved mechanically like a spring through the Euler-Bernoulli equation [1] at the frequencies we probed (as confirmed by oscillating the cantilever without material load). We could thus assume a spring constant $k$ relating static load $P$ and cantilever displacement $d$ through $P = k \cdot d$. The bending stiffness $k$ was calibrated using a high-precision scale (Cubis microbalance, Sartorius AG) and the radius of the bead was measured using an optical microscope (BX60, Olympus). For every sample, we first performed indentations to determine the linear elastic deformation regime. To that end we translated the probe with the piezoelectric actuator resulting in an indentation depth $h$ and cantilever deflection $d_{cantilever}$ upon contact with the sample:

$$d_{piezo} = h + d_{cantilever} \tag{1}$$

Linear piezoelectric movement leads to non-linear load $P$ and indentation depth $h$, since these quantities are related by Hertzian theory [2, 3] through:

$$P = k \cdot d_{cantilever} = \frac{8}{3}\sqrt{R}\frac{G}{1-\nu}h^{3/2} = \frac{8}{3}\sqrt{R}\frac{G}{1-\nu}(d_{piezo} - d_{cantilever})^{3/2} \tag{2}$$

where $R$ denotes the radius of the spherical indenter, $G$ the material shear modulus and $\nu$ the Poisson ratio. We used these results under linear elastic assumptions to determine which load $P$ and indentation depth $h$ were within the linear (visco-)elastic limit for our dynamic load-sweep. Typically, the sample was indented to a depth of 3-8 $\mu$m, with loads ranging over 3 orders of magnitude from 0.1-100 $\mu$N depending on material stiffness. For samples with different stiffnesses, appropriate probes with $k$ = 0.5-40 N/m were used to maximize sensitivity to cantilever deflection and indentation. To quantify the stiffness gradient samples we used cantilevers with a bending stiffness $k$ = 6-8 N/m.

# 2 Feedback error signal

As the dynamic sweep was performed, the actual cantilever deflection from phase unwrapping was determined at every time point. The subsequent mismatch between the load (given by cantilever deflection) and the sweep load produced an error signal:

$$e(t) = P_{sweep}(t) - k \cdot d_{cantilever}(t) \tag{3}$$

The error signal controlled a direct feedback loop with action onto the piezoelectric extension. Continuous adjustment over time made sure the actual load onto the sample followed the predefined load sweep. Finally, our readout was the indentation depth as given by $h(t) = d_{piezo} - d_{cantilever}$ dependent on the piezoelectric adjustment and the cantilever deflection. The piezoelectric displacement was monitored through the strain gauge while the cantilever displacement was measured by live demodulation, as explained in the main text.

For the mapping of a surface with a large variation in stiffness, load-controlled indentations may cause the indentation depth to become too large for the theoretical limit of small deformations relative to probe size (i.e. $h << R$). To ensure small deformations and probing at similar depth when measuring samples with a heterogeneous stiffness, we controlled the feedback onto the piezoelectric actuator relative to an indentation depth sweep. The indentation depth is given by $h = d_{piezo} - d_{cantilever}$. Note here that especially for highly heterogeneous samples the cantilever deflection can be very large relative to the piezoelectric movement leading to a small indentation depth (over 2 orders of magnitude from typically 200 nm to 20 $\mu$m, see figure 3 in the main text). To correct for that more piezoelectric movement is needed to obtain the same indentation depth as in a softer location. The error signal for indentation-controlled movement thus becomes:

$$e(t) = h_{sweep}(t) - h(t) = h_{sweep}(t) - (d_{piezo}(t) - d_{cantilever}(t)) \tag{4}$$

Further analysis to determine the storage and loss modulus was fully analogous to a load-controlled indentation sweep and is described in the main text.

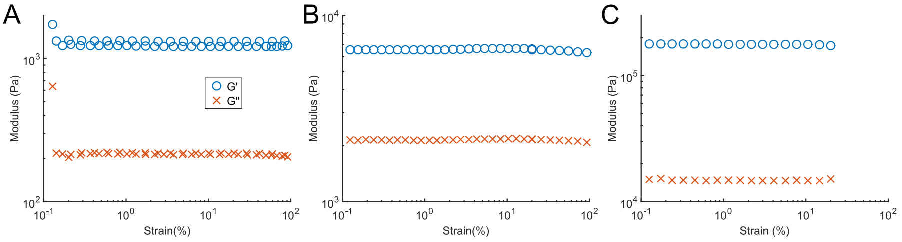


**Figure S1:** The silicone polymers under investigation behave as linear viscoelastic materials over the strain range probed by macroscale rheometry and indentation. Up to large shear strain ($\gamma > 20\%$), the LS- (A), MS- (B) and HS samples (C) show no change in $G'$ and $G''$. Data are shown for $f = 1$ Hz, but linear viscoelasticity still holds for the full frequency range $f = 0.01 - 10$ Hz.

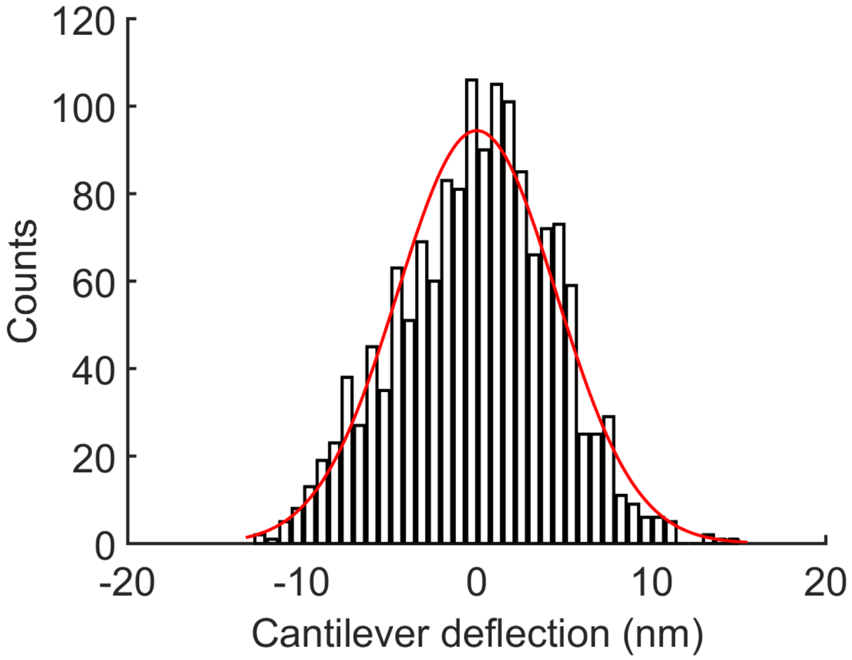


**Figure S2:** Precision of the cantilever deflection measurement. A Gaussian fit (red line) to a distribution of zero cantilever deflections gives the precision of deflection measurement $\sigma_{cantilever} = 5$ nm. Cantilever deflection precision relates to the precision of force measurement dependent on cantilever stiffness $k$ through $\sigma_P = k \cdot \sigma_{cantilever}$.

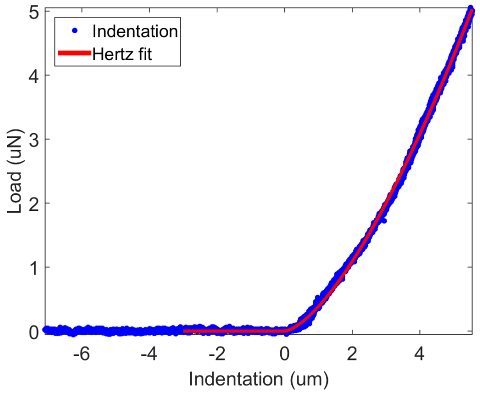


**Figure S3:** A Hertzian fit (equation 2 in supporting information) is made to the indentation approach curve to determine the exact point of contact. Over the full indentation approach (blue) a Hertzian fit (red line) works well since we are in the linear elastic regime.

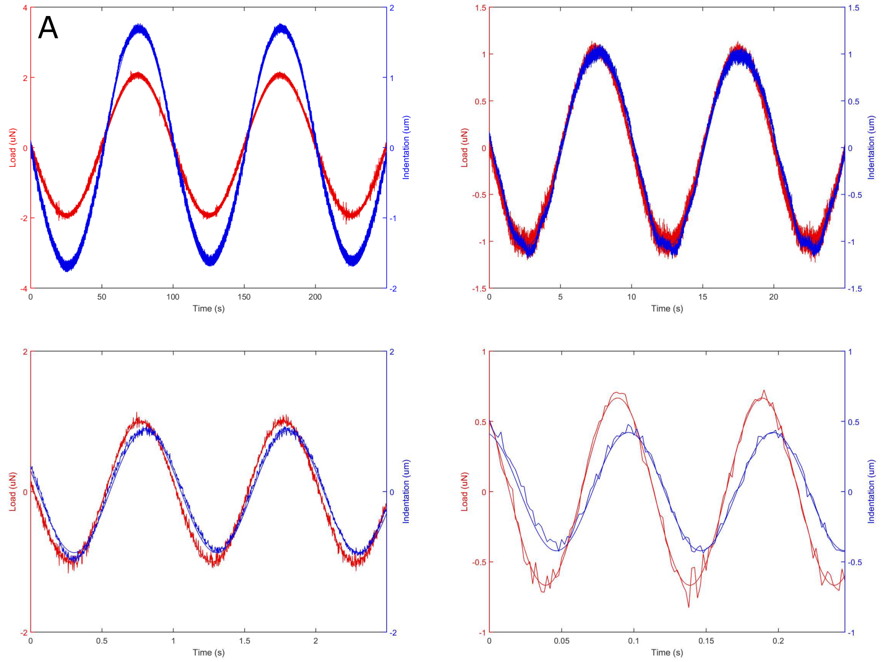


**Figure S4:** Sinusoidal fits to oscillations on a MS sample, corresponding to figure 4C in the main text. At f= (A) 0.01 Hz, (B) 0.1 Hz, (C) 1 Hz and (D) 10 Hz, load (red) and indentation (blue) are depicted. Note that from A-D with increasing frequency; (1) the amplitude of the indentation response decreases, indicating an increase in stiffness and (2) the phase shift between load and indentation increases indicating a more viscous response. These characteristics are captured by (1) the absolute increase of both $G'$ and $G''$ and (2) the relative increase of $G''$ relative to $G'$, respectively.

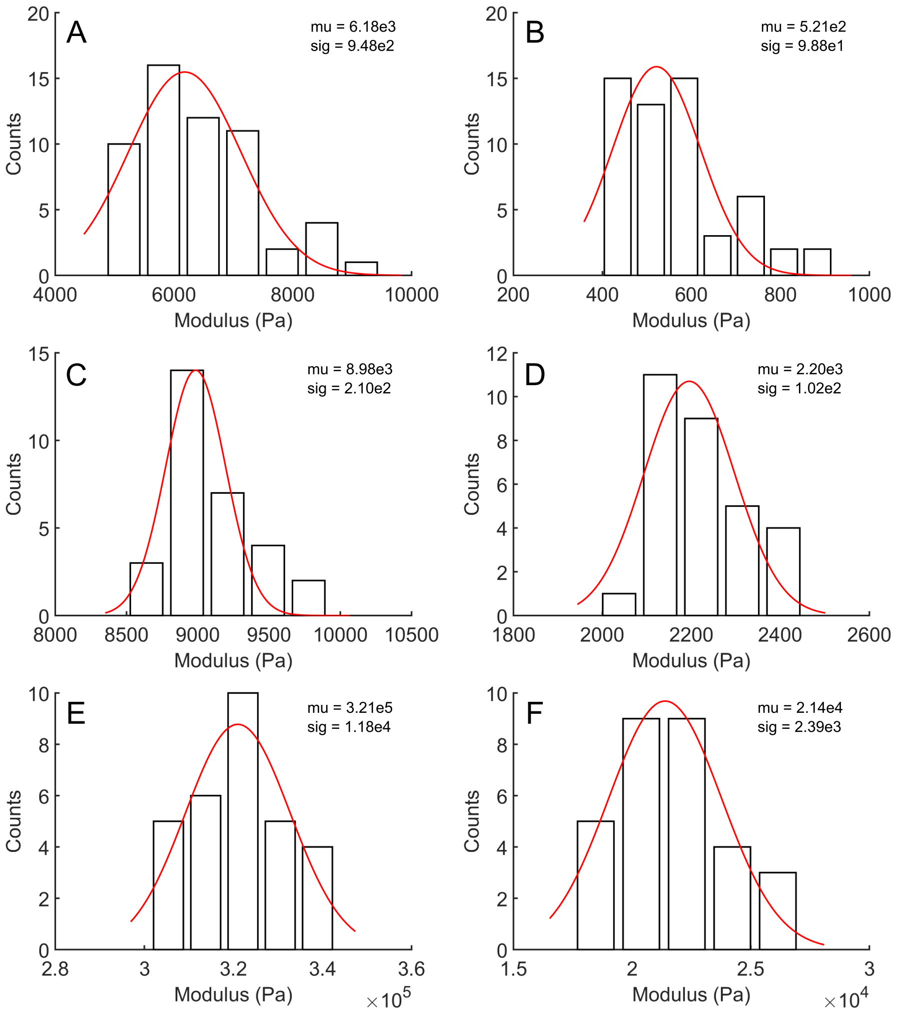


**Figure S5:** Precision of $G'$ and $G''$ as probed at $f = 1$ Hz. Storage and loss moduli $G'$ and $G''$ from repeated indentations on the same spot on (A-B) a LS sample, (C-D) a MS sample and (E-F) a HS sample. Distributions of results are fitted with a Gaussian distribution (red line) and the mean and standard deviation are depicted. The precision of the measurement of $G'$ and $G''$ thus typically has a standard deviation of $\sim$ 5-15 % of the modulus which characterizes the measurement precision.

# References

[1] Roy R Craig and Andrew J Kurdila. *Fundamentals of structural dynamics.* John Wiley & Sons, 2006.

[2] Kenneth L Johnson. *Contact Mechanics.* Cambridge University Press, Cambridge, 1985.

[3] Heinrich Hertz. Über die berührung fester elastischer körper. *Journal für die reine und angewandte Mathematik*, 1882.
